# Real-time video communication between ambulance paramedic and scene – a simulation-based study

**DOI:** 10.1186/s12913-022-08445-w

**Published:** 2022-08-17

**Authors:** Roman Sonkin, Eli Jaffe, Oren Wacht, Helena Morse, Yuval Bitan

**Affiliations:** 1 Community Division, Magen David Adom, Ha-Plada 5, 6021805 Or-Yehuda, Israel; 2 Department of Emergency Medicine, Faculty of Health Sciences, Ben-Gurion University of the Negev, Beer-Sheva, Israel; 3 Department of Health Policy and Management, Faculty of Health Sciences, Ben-Gurion University of the Negev, Beer-Sheva, Israel

**Keywords:** EMS, Emergency Medical Services, MDA, Magen David Adom, Resuscitation, Telemedicine, Teletriage

## Abstract

**Introduction:**

Telemedicine has been widely used in various medical settings including in Emergency Medical Services (EMS). The goal of this study was to assess the possible roles of real-time video communication between paramedics and bystanders at scenes of emergency, in the analysis and treatment of patients.

**Methods:**

44 experienced paramedics participated in a simulation. Participants communicated with the experimenter presenting video clips showing patients that simulated three emergency scenarios: trauma, an unresponsive patient with cardiac arrest, and an opiate overdose. The simulation sessions were conducted through Zoom™, recorded, and then analyzed to document participants’ questions, requests, instructions, and their timings during each scenario.

**Results:**

The trauma scenario was assessed most promptly, with instructions to handle the bleeding provided by all paramedics. In the unresponsive patient with cardiac arrest scenario, most of the participants achieved a correct initial diagnosis, and in the opiate overdose scenario over half of paramedics sought visual clinical clues for the differential diagnoses of loss of consciousness and their causes. Additional results show the type of assessment, treatment and diagnosis participants provided in each scenario, and their confidence about situation.

**Conclusions:**

The findings show that direct video communication between paramedic and scene may facilitate correct diagnosis, provision of instructions for treatment, and early preparation of medications or equipment. These may decrease time to correct diagnosis and lifesaving treatment and impact patient morbidity and mortality. Moreover, the findings highlight the difference between incidents with higher visual clarity, such as trauma, and conditions that require an extended diagnosis to reveal, such as unresponsive patients. This may also increase the paramedics’ mental preparedness for what is expected at the scene.

## Background

Emergency Medical Services (EMS) are an integral part of any effective and functional healthcare system, as defined by the World Health Organization (WHO) [1, 2]. It has been found that faster response and treatment is correlated with higher survival rates in certain medical emergencies [3, 4]. Thus, to provide emergency care, it is imperative to seek effective and efficient ways to decrease the time before patients receive emergency treatment. This study aims to explore the role of real-time video communication between paramedics that are making their way to the scene and bystander at the scene in the analysis and treatment of patients in emergency situations. To the extent of this literature review, this was the first study to explore this field by utilizing simulated video calls between paramedics in a stationary room and the simulated scene.

EMSs’ mainstay role is to respond quickly and efficiently through two main vectors – firstly, rapid response, tele-triage and first aid guidance by the emergency medical dispatcher at the call center; and secondly, dispatching first responders and ambulance teams to provide urgent medical care and transport to a suitable hospital [5–7].

Telemedicine has been used reliably to support decision-making and action in various medical settings, such as remote consultations with specialists in areas where they are not readily available, with high diagnostic accuracy [8, 9]. Current technologies allow voice and video telemedicine to be highly cost-effective [10], using the patient or medical staff member’s own telephone device [11]. New models of out-of-hospital care are being developed and investigated to improve patient care; Abrashkin, et al. (2020) found that emergency department physicians using video communications reported enhanced clinical evaluation of the patients compared with telephone communication alone [12].

Traditionally, communications with bystanders at the scene are in the realm of the emergency medical dispatcher’s responsibilities. Current literature points to an increase in survival rates in cardiac arrest situations with dispatcher-assisted bystander cardio-pulmonary resuscitation (CPR), and an improvement in the overall efficacy of lay-person CPR [13, 14]. Furthermore, it has been found that CPR in video settings where paramedics can give live feedback to the bystander is more effective than telephone or video-instructed CPR alone [15].

The primary objective of this study was to assess the implications of a video communication between the bystanders at the scene and paramedics on their way to the scene. This study evaluated the effectiveness and advantages of this new form of telemedicine among three types of emergency medical scenarios – trauma, an unresponsive patient with cardiac arrest, and an unresponsive patient with opiate overdose. These medical scenarios vary in the visual representation of the sickness or injury, as well as in the appropriate response. Penetrating trauma is commonly characterized by acute hemorrhaging and can be diagnosed by gross examination of injuries [16]. On the other hand, diagnosis of cardiac arrest with an unresponsive patient requires clinical cues such as pulse, respiration, and assessment of consciousness [17]. Moreover, an opioid overdose, with clinical symptoms including constricted pupils, respiratory depression, and decreased level of consciousness, requires more advanced analysis [18].

The primary aim of this study was to show that video communication could facilitate quick and correct diagnosis, allow for provision of lifesaving treatment instructions while helping the paramedics feel prepared and confident about what they are going to find at the scene. For the secondary aim of this study we defined the term “visual clarity” as the exposure level of important medical condition features that are visible and can be easily viewed, and compared medical conditions with higher visual clarity (, such as trauma, and situations with lower visual clarity, such as opiate overdoses and unresponsive patients.

## Methods

### Study design and setting

This was a low-fidelity simulation, aimed at making the paramedics participating in the study feel as close as possible to real-life scenarios. The scenarios were dynamic with prepared responses to different types of requests and questions that may arise to both the correct diagnosis and other diagnoses on the differential. This study followed the STROBE guidelines and its simulation in healthcare extensions [19, 20]. In this simulation, participants could communicate with a bystander at the scene, ask questions, and see video from the scene from the viewpoint of a bystander’s phone. To achieve consistency in the scenarios across all repetitions, video clips were recorded in advance to imitate the bystander’s answers to various questions from the paramedics. Thus, the participants had requests and questions, and the experimenters showed video clips providing consistent answers. Although this method was limited by the experimenters’ ability to answer some of the paramedics’ questions, it simulated real-life callers who will not always have answers or be willing to comply with specific requests. However, the experimenters were prepared to answer questions and requests that were not anticipated by using their judgment and replying either “I don’t know”, “I can’t” or “I am afraid”, they were instructed to be consistent with the same answer to the same request.

The clinical scenarios were filmed using a Sony™ video camcorder at a real residential apartment by human factors engineering students and their supervising lecturer, in on-scene consultation with two senior paramedics. The simulated patient was a medical actor, decorated by a professional medical moulage artist.

This study was approved by the Institutional Review Board of Ben-Gurion University of the Negev (BGU) and the scientific committee of Magen David Adom (MDA), Israel’s national EMS organization. Patient and public were not involved in the study design, outcome measures or recruitments.

### Scenarios

Three clinical scenarios were composed by senior paramedics, following their characteristic clinical pictures: (1) unresponsive patient with cardiac arrest – acute coronary syndrome (ACS), quickly deteriorating to a gasping pulseless patient requiring CPR [21–23]; (2) opiate overdose – non-prescribed use to treat unbearable chronic pain, causing altered mental status and respiratory depression [24, 25]; and (3) hemorrhagic shock – deep injury caused by an electric tool leading to active bleeding and hypovolemic shock [26, 27]. An appropriate medical interview checklist was composed, and possible differential diagnosis questions and appropriate answers were prepared [28].

### Experiment conduction

The experiment was conducted during April 2020, through Zoom™ conferencing platform at the pre-scheduled time initiated by the experimenter. Participants were instructed to be at a comfortable and non-noisy location at the time of the experiment and to use only their smartphones for the purpose of the video call. The recorded scenarios were put into a Microsoft Power-Point presentation to allow for seamless switching between them during a video call and were broadcast by OBS Studio software as a webcam feed. After an introduction and obtaining informed consent, each participant completed three semi-interactive scenarios that were presented in a counterbalanced order across subjects. The experimenters would present video clips showing the answers to questions asked by the participants. The participants responded to a few survey questions about diagnosis, treatment and their feelings following each scenario and a concluding survey in the end of the experiment about their point of view on the whole subject of video communication between the scene and the paramedic.

### Experimenters

Two engineering students served as experimenters of this study. Both did not undergo first aid or CPR training in the 5 years prior to the experiment, thus were not able to affect the participants’ clinical assessment.

### Participants and recruiting

Forty-four participants were recruited through a post shared on social media in groups of MDA paramedics. They received a breakfast voucher (value of ~ 13 USD) as a token of appreciation for their participation. The collected data was deidentified and cannot be linked to the participant.

### Inclusion criteria

Active paramedics at MDA ambulance teams at the time of the study.

### Exclusion criteria

Scenarios where an improper device was used (e.g., computer or tv screen) or scenarios with technical difficulties rendering them unsuitable for analysis.

### Likert scale questionnaire

At the end of each scenario, the participants answered a questionnaire. The Likert scale (1–7) questions were shown to the participants as − 3 to + 3: “Influence of video on your feeling of confidence?” (− 3 reduced, 0 no influence, + 3 added), “Influence of video on your perception of the clinical situation at scene” (− 3 interrupted, 0 no influence, + 3 aided), “Influence of video on your feeling of personal and mental preparedness” (− 3 reduced, 0 no influence, + 3 improved), “Level of influence the video will have in real life on morbidity and mortality” (− 3 increase, 0 no influence, + 3 reduce).

The likert scale (1–5) questions were described from 1-non at all to 5-very much/high: “Rank the difficulty in a achieving a diagnosis through video communication”, “Rank the ability to remotely instruct the caller through video communication”, “Rank the ability to establish cooperation with the caller”, “Rank the ability to establish trust between the caller and paramedic”, “Rank the positive influence of early establishment of cooperation and trust on the progression of the incident”, “Rank the feasibility of video calling for medical interview and physical diagnosis”.

#### Data analysis

Each session was recorded and then analyzed by a different experimenter than the one who conducted the recorded experiment. An experienced paramedic assisted in resolving conflicts and uncertainties. The analysis included the evaluation participants’ questions, instructions and their timings compared to a medical interview checklist which was compiled for each scenario. Demographic information and questionnaire answers were then assembled and compared between scenarios.

## Results

### Demographics

Of the 43 valid participants, 30 (70%) were male. Participants’ mean tenure as paramedics was 5 years (range 1–20). Of the participants, 32 (74%) were team leaders on ambulances, with their mean tenure as team leaders being 4.5 years (range 0.8–8), and 10 (23%) had an EMS dispatcher qualification.

### Analysis of the scenarios

One participant was excluded from the study for using a computer screen for the video call, all three scenarios were not analyzed. In addition, one gasping scenario and one opiate overdose scenario were excluded for technical difficulties rendering them unsuitable for analysis.

### Assessment

In the trauma scenario, all the participants referred to the bleeding within a median of 55 seconds (IQR 00:32–01:40), assessed skin tone in 7 (16%) sessions within a median of 3 minutes and 22 seconds (IQR 2:49–3:50) and asked to see the patient’s face in 6 (14%) sessions within a median of 3 minutes and 6 seconds (IQR 02:08–03:56).

In the cardiac arrest scenario, the participants assessed the level of consciousness (LOC) in 41 (98%) sessions within a median of 1 minute and 24 seconds (IQR 00:41–02:33), evaluated breathing in 37 (88%) sessions within a median of 1 minute and 39 seconds (IQR 00:54–03:14). The participants instructed the bystander to check central pulse in 10 (24%) sessions within a median of 3 minutes and 58 seconds (IQR 03:06–05:20) - these participants asked to measure the pulse only after the patient collapsed. Skin tone and sweating were assessed in 19 (45%) sessions within a median of 1 minute and 45 seconds (IQR 01:06–02:28) and a list of patient’s current medications was requested in 10 (24%) sessions within a median of 2 minutes and 38 seconds (IQR 01:25–03:17).

In the opiate overdose scenario, LOC was assessed in 39 (93%) sessions within a median of 51 seconds (IQR 00:23–01:38), breathing was evaluated in 38 (90%) sessions within a median of 47 seconds (IQR 00:31–01:12). The participants instructed the bystander to check central pulse in 14 (33%) sessions within a median of 2 minute and 18 seconds (IQR 00:55–03:32). Skin tone and sweating were assessed in 20 (48%) sessions within a median of 3 minutes and 15 second (IQR 01:58–04:13), pupils were assessed in 22 (52%) sessions within a median of 3 minutes and 45 seconds (IQR 02:01–05:00). A list of patient’s current medications was requested in 23 (55%) sessions within a median of 3 minutes and 54 seconds (IQR 02:19–05:18). Participants inquired about diabetes and a glucometer in 29 (69%) sessions within a median of 2 minutes and 37 seconds (IQR 01:30–03:33).

### Diagnosis

In the trauma scenario all the participants achieved correct diagnosis immediately while in the cardiac arrest scenario the participants achieved first diagnosis within a median of 1 minute and 23 seconds (IQR 00:35–02:09) after the patient collapsed. In 35 (83%) sessions the first diagnosis was correct (cardiac arrest), and in 7 (17%) sessions the first diagnosis was myocardial infarction (MI) (4 of which changed their diagnosis to cardiac arrest within a median of 58 seconds IQR 03:00–01:43 after the patient collapsed. In the opiate overdose scenario, 33 participants achieved first diagnosis within a median of 4 minutes and 34 seconds (IQR 02:52–05:26). In 24 (57%) sessions the first diagnosis was correct (opiate overdose), and in 7 (17%) sessions diagnosis the first diagnosis was stroke (3 of which changed their diagnosis to opiate overdose within a median of 6 minutes and 27 seconds IQR 05:25–06:58 of the beginning of the session.

### Treatment

In the trauma scenario, all participants requested to apply stronger pressure on the wound within a median of 42 seconds (IQR 00:27–01:16). They requested to replace the bandage in 38 (88%) sessions within a median of 54 seconds (IQR 00:30–02:24). The participants provided instructions to improvise a tourniquet in 32 (74%) sessions within a median of 1 minute and 18 seconds (IQR 00:49–02:00).

In the cardiac arrest scenario, the participants requested that the patient be lowered to the floor in 41 (98%) sessions within a median of 38 seconds (IQR 00:12–01:06) after the patient collapsed. Instructions to provide chest compressions were given in 38 (90%) sessions within a median of 01:21 seconds (IQR 00:40–01:55) after the patient collapsed. In 35 (83%) sessions feedback on the quality of chest compressions was provided within 45 seconds (IQR 00:32–01:24) after chest compressions were initiated.

In the opiate overdose scenario, the participants discovered opiate patches in 23 (55%) session, and the caller was instructed to remove them within a median of 5 minutes and 6 seconds (IQR 04:11–06:05) from the beginning of the session.

### Safety

The trauma scenario was unique in its concern for the patient and bystander safety as the dangerous machine that caused the injury was still working, as opposed to the other two scenarios where there were no safety concerns to address. Thus, safety concerns were addressed in 16 (37%) sessions within a median of 2 minutes and 35 seconds (IQR 01:45–03:22).

### Survey questions

On a Likert scale of 1–7, the participants ranked the influence of the video on their feeling of confidence at a mean of 5.96 (SD = 1.41), their perception of the situation in the scene at a mean of 6.24 (SD = 1.32), and their feeling of personal and mental preparedness at a mean of 6.12 (SD = 1.35). In addition, the participants marked that they believe the video will influence morbidity and mortality at a mean of 5.52 (SD = 1.54). In 97 (75.78%) cases the video caused the participants to modify the team briefing from the briefing they would have given based on the two-three lines of case description as they receive in real life. Of these, 48 (49.48%) would assign specific roles and 61 (62.89%) would prepare specific equipment for the scenario. In terms of difficulty achieving a diagnosis through the video call, 49 (38.28%) of the participants reported no difficulty at all.

On a Likert scale of 1–5, the participants ranked the difficulty in a achieving a diagnosis through video communication at a mean of 1.58 (SD = 1.65), the ability to remotely instruct the caller was ranked at a mean of 3.47 (SD = 1.41). The participants ranked the ability to establish cooperation at a mean of 3.72 (SD = 1.26), The ability to establish trust between the caller and paramedic was ranked at a mean of 3.75 (SD = 1.19). Positive influence of early establishment of cooperation and trust on the progression of the incident was ranked at a mean of 4.12 (SD = 1.09). Finally, the feasibility of video calling for medical interview and physical diagnosis was ranked by the participants at a mean of 3.91 (SD = 1.11).

## Discussion

In the effort to discover more ways to improve pre-hospital care, it is imperative to find innovative ways to improve emergency care at the scene. This study examined paramedics’ telemedicine communications before arriving at the scenes of three scenarios: a trauma, an unresponsive patient with cardiac arrest, and an opiate overdose. The paramedics did not undergo any specific telemedicine related training before the experiment and were instructed by their clinical experience and standard EMS protocols.

The time to make a first diagnosis, and time to make a correct diagnosis, were significantly different between the three scenarios. These finding highlights that when using telemedicine there is a difference between emergency situations with higher visual clarity, such as trauma, and life-threatening conditions that require profound diagnosis to reveal, such as an unresponsive patient with cardiac arrest or opiate overdose. The trauma was assessed most quickly of the three. In the trauma scenario, all paramedics provided the correct instructions. The paramedics were able to instruct and mobilize the bystander to take actions to stop or slow the bleeding. This finding points to the potential life-saving benefits of telemedicine in pre-hospital settings, particularly in cases with active bleeding. These findings support the secondary aim showing that a paramedic can make a diagnosis and give instructions through telecommunication with the bystander, particularly in cases with high visual clarity. However, paramedics’ safety concerns might be low due to the mental distance of the paramedic from the scene and their focus on hemorrhage control.

In the unresponsive patient with cardiac arrest scenario, the majority of the participants were correct in their initial diagnoses. The remaining participants initially diagnosed myocardial infarction, but over half of those that misdiagnosed changed their diagnosis to cardiac arrest. This finding also demonstrates the secondary aim, showing that the video call between a paramedic and scene may facilitate diagnosis within a short period of time, although further research is required in the clinical setting to strengthen the evidence. Most paramedics identified that the patient’s breathing was ineffective, and instructions for CPR were provided, despite the caller’s answer that the patient was breathing (non-effective agonal respiration). These findings point to the benefits of video telecommunication with bystanders, allowing paramedics to better understand a scenario through visual cues, such as seeing a patient’s state and breathing, and provide instructions accordingly, rather than solely relying on a bystander’s assessment. Current studies show that video supervision of lay-person CPR is superior to instruction via audio-call, our findings although in a simulated environment also support these results [29]. To improve their assessment paramedics used additional video-specific actions such as a close-up of the patient’s face, visually see the patient’s chest movements to assess breathing, visually see the patient’s medication, and see the patient’s skin tone. The overall scenario time and diagnosis time in the cardiac arrest scenario was longer than the trauma scenario, which coincides with the nature of the scenario’s visual clarity.

In the opiate overdose scenario, over half of paramedics sought visual clinical clues for the differential diagnoses of loss of consciousness and their causes. Visual cues were utilized through close-ups of the patient’s pupils, assessing skin tone and sweating, requesting to see medications in the patient’s surroundings, and detecting patches. As the scenario with the lowest visual clarity this was the most difficult scenario to diagnose through telecommunication, but the paramedics proved that they could improvise and find innovative ways to get to the right diagnosis.

The study revealed that the real-time video communications between the scene and paramedic allow for greater confidence for the paramedic before arrival to the scene. Furthermore, paramedics reported that the video influenced their mental and physical preparation, as well as their perception of the scene.

This study demonstrates that experienced paramedics can use real-time video communication to facilitate quick and correct diagnosis of three life threatening medical emergencies. These findings might lead to changes in the paramedics’ treatment protocols, utilizing such communication to provide instructions for treatment and respond to any changes in the patient’s condition during the time between the call for help and the ambulance’s arrival at the scene. Such communication would also allow the paramedics arriving at the scene with more information and familiarity with the scenario and the environment.

Future studies should focus on establishing guidelines and working protocols for such telemedicine work procedure, testing them in high-fidelity simulation scenarios and comparing them to existing treatment protocols. This development might lead the way to important changes in the way EMS are supporting patients before the team arrival at the scene.

### Limitations

As a low-fidelity simulation study, this experiment was conducted in a “sterile”, static, and quiet environment, which differs from the paramedics’ working environment on their way to the scene that involves movement, noise, and other stressors.

## Conclusions

In summary, emergency medical dispatchers at the call center have limited time to understand the situation based on a short interview, and the information that the ambulance team receive is brief, limited and second hand [30]. As shown in this study, direct real-time video communication between ambulance paramedics and the scene may facilitate conditions to establish patient-caregiver rapport, achieve correct diagnosis, and provide instructions for lifesaving treatment by the person who arrives at scene, all before the team arrives at the scene. However, further research in the real working environment is needed before this is implemented. Clear protocols should also be made available to cover major topics such as addressing safety concerns. This communication method may also allow paramedics to adequately prepare for response before arriving at the patient’s location.

## Data Availability

The datasets used and/or analysed during the current study available from the corresponding author on reasonable request.

## Abbreviations

EMS: Emergency Medical Services
WHO: World Health Organization
CPR: Cardiopulmonary Resuscitation
BGU: Ben Gurion University
MDA: Magen David Adom
ACS: Acute Coronary Syndrome
MI: Myocardial Infarction
